# Unraveling the structural elements of pH sensitivity and substrate binding in the human zinc transporter SLC39A2 (ZIP2)

**DOI:** 10.1074/jbc.RA118.006113

**Published:** 2020-08-26

**Authors:** Gergely Gyimesi, Giuseppe Albano, Daniel G. Fuster, Matthias A. Hediger, Jonai Pujol-Giménez

**Affiliations:** ‡ Institute of Biochemistry and Molecular Medicine, University of Bern, Bühlstrasse 28, 3012 Bern, Switzerland; § National Center of Competence in Research, NCCR TransCure, Bühlstrasse 28, 3012 Bern, Switzerland; ¶ Department of Nephrology and Hypertension, Inselspital, Bern University Hospital, University of Bern, Freiburgstrasse 18, 3010 Bern, Switzerland

**Keywords:** zinc, membrane transport, homology modeling, structure–function, site-directed mutagenesis, metal homeostasis, SLC, ZIP

## Abstract

The transport and ion-coupling mechanisms of ZIP transporters remain largely uncharacterized. Previous work in our laboratory has revealed that the solute carrier family 39 member A2 (SLC39A2/ZIP2) increases its substrate transport rate in the presence of extracellular H^+^. Here, we used a combination of *in silico* and *in vitro* techniques involving structural modeling, mutagenesis, and functional characterization in HEK293 cells to identify amino acid residues potentially relevant for both the ZIP2–H^+^ interaction and substrate binding. Our ZIP2 models revealed a cluster of charged residues close to the substrate–translocation pore. Interestingly, the H63A substitution completely abrogated pH sensitivity, and substitutions of Glu-67 and Phe-269 altered the pH and voltage modulation of transport. In contrast, substitution of Glu-106, which might be part of a dimerization interface, altered pH but not voltage modulation. Substitution of Phe-269, located close to the substrate-binding site, also affected substrate selectivity. These findings were supported by an additional model of ZIP2 that was based on the structure of a prokaryotic homolog, *Bordetella bronchiseptica* ZrT/Irt-like protein (bbZIP), and *in silico* p*K_a_* calculations. We also found that residues Glu-179, His-175, His-202, and Glu-276 are directly involved in the coordination of the substrate metal ion. We noted that, unlike bbZIP, human ZIP2 is predicted to harbor a single divalent metal-binding site, with the charged side chain of Lys-203 replacing the second bound ion. Our results provide the first structural evidence for the previously observed pH and voltage modulation of ZIP2-mediated metal transport, identify the substrate-binding site, and suggest a structure-based transport mechanism for the ZIP2 transporter.

## Introduction

Zinc (Zn^2+^) is a transition metal required in mammalian cells as a cofactor and structural component of a wide variety of cellular proteins, as well as a signaling molecule. Consequently, intracellular Zn^2+^ bioavailability is essential for various cellular components and signaling pathways necessary for the survival and growth of mammalian cells. To ensure an adequate control of intracellular Zn^2+^ levels, cells possess specific transport proteins on both plasma and organellar membranes as well as specialized storage and carrier proteins in the cytoplasm (1). Zn^2+^ transporter proteins are grouped into two solute carrier families, SLC39 and SLC30, that differ in terms of the direction of metal ion transport. SLC39 transporters accumulate Zn^2+^ into the cytoplasm (*i.e.* import from the extracellular medium and export from intracellular organelles), whereas SLC30 transporters mobilize Zn^2+^ out of the cytoplasm in the opposite direction (2). The SLC39 family, also known as ZIP (Zrt-, Irt-like proteins), is constituted by 14 different members, which are subdivided into subfamilies I, II, LIV-1, and gufA, according to their sequence similarities (3). To date, there is no direct structural information available for any full-length mammalian ZIP transporter. However, all the ZIP members are predicted to have eight transmembrane domains, with their N and C termini facing the extracellular space (1, 4). Interestingly, recent computational studies using co-evolution contact predictions and Rosetta *ab initio* structure prediction generated the first molecular model for a mammalian ZIP transporter (5). This model of human ZIP4, a member of the subfamily LIV-1, exhibits eight transmembrane helices (TMH),^6^ in which TMHs 2, 4, 5, and 7 form a helical bundle containing the putative metal-coordination site. Alanine replacement of the histidine and aspartic acid residues present in the proposed metal-coordination site altered the Zn^2+^ transport kinetics, validating this structural model (5). The described metal-coordination site resembles that of the crystal structure of the SLC30 family homolog YiiP (6) (*i.e. Escherichia coli* Zn^2+^/H^+^ antiporter), suggesting a common structural element for the different Zn^2+^ transporters. Another interesting aspect revealed by these studies is that the predicted contacts used as a basis to build the model could only be observed when ZIP4 was modeled as a dimer in which the TMHs form the dimer interface (1, 5). This finding is supported by previous evidence of homodimerization of other ZIP transporters, such as ZIP2, ZIP7, and ZIP13, or the heteromer of ZIP6 and ZIP10 (1). Although not included in the computational model, ZIP transporters are predicted to possess a large cytoplasmic loop between TMHs 3 and 4, which contains a variety of zinc-coordination sites likely functioning as a regulatory region that senses and responds to changes in intracellular Zn^2+^ concentration (1). Recently, the first experimental evidence of the overall structure emerged through the crystallization of a prokaryotic ZIP homolog, bbZIP, showing remarkable overall agreement with the previously described theoretical model (7). Based on the X-ray structure, a structural model of the human ZIP4 protein was derived, and several structural elements of the protein, such as the substrate-binding site, have been identified (7).

In terms of the functional properties of the SLC39/ZIP family, members of this family transport a wide variety of divalent metals in addition to Zn^2+^, including iron (Fe^2+^), manganese (Mn^2+^), copper (Cu^2+^), and cadmium (Cd^2+^) (1). Even though the transport mechanism of ZIPs is not yet fully understood, it has been reported that the transport process is not dependent on ATP hydrolysis (8, 9) and, for some of the ZIP transporters such as ZIP2, ZIP8, and ZIP14, a Zn^2+^/HCO_3_^−^ symport mechanism has been proposed (8, 10, 11). In contrast, studies conducted with a bacterial ZIP transporter suggested that this protein might function as an ion-selective channel in which Zn^2+^ is incorporated into the cytoplasm via an electro-diffusion mechanism (12).

SLC39A2, also known as ZIP2, belongs to the ZIP subfamily II, and it was cloned and functionally characterized for the first time by Gaither and Eide in the year 2000 (8). In that work it was described that ZIP2-overexpressing K562 cells (human immortalized myelogenous leukemia cell line) accumulate Zn^2+^ in a time-, temperature-, and concentration-dependent manner, that the transport process was independent of ATP hydrolysis as well as Na^+^ and K^+^ gradients, and was stimulated in the presence of extracellular HCO_3_^−^ but inhibited by lowering the extracellular pH. Altogether, these findings indicated that ZIP2 behaved as a Zn^2+^/HCO_3_^−^ symporter (8). In contrast, prior work conducted in our laboratory suggested that ZIP2 activity is stimulated rather than inhibited by the presence of H^+^ (13). In a more recent study, we showed that ZIP2-mediated transport in both microinjected *Xenopus laevis* oocytes and transiently transfected HEK293 cells is independent of both HCO_3_^−^- and H^+^-driving forces, but modulated by extracellular pH and voltage (14). However, structural details of how H^+^ affects transport activity are still missing and remain to be described.

Originally, ZIP2 expression was detected in uterus and prostate (8), in the latter also at the protein level (15). In prostate cancer cells, ZIP2 is down-regulated to decreased zinc levels, thereby restoring m-aconitase 2 activity and promoting ATP production via the tricarboxylic acid cycle (15,16, 17, 18, 19,20). Similarly, low zinc levels and increased ZIP2 expression have also been linked to pulmonary inflammatory diseases (21, 22). ZIP2 also seems to play a role upon zinc depletion. Cao *et al.* (23) found that zinc depletion in peripheral blood mononuclear cells and the THP-1 monocytic cell line triggered up-regulation of ZIP2 with the concomitant down-regulation of zinc-binding metallothioneins. Later studies also found ZIP2 expression in macrophages, where it was the most responsive gene upon zinc depletion (24). Recently, in healthy human skin samples, Inoue *et al.* (25) discovered that ZIP2 is up-regulated upon the induction of differentiation in cultured keratinocytes. Interestingly, ZIP2 knockdown inhibited the differentiation of keratinocytes and consequently the formation of a three-dimensional cultured epidermis. Studies with ZIP2–KO mice did not reveal any specific phenotype. However, these mice were more susceptible to abnormal embryonic development because of zinc deficiency during pregnancy (26). Taken together, these physiological roles warrant a more detailed investigation of the transport and ion-coupling mechanism of subfamily II ZIP transporters, of which ZIP2 is the most well-characterized member.

In this work, taking advantage of the first available human ZIP4 model (5), we developed a homology-based model for human ZIP2 and proposed residues that might play a role in both Zn^2+^-coordination and H^+^-mediated modulation of the transport process. Site-directed mutagenesis of the proposed amino acid residues followed by detailed functional characterization of mutants validated the proposed model and provided the first structural details of the Zn^2+^-coordination site and H^+^ modulation of the transport process mediated by human ZIP2. In addition, through comparison of our model with another model built based on the bbZIP structure, we propose a transport mechanism for H^+^-sensitive transport of divalent metal ions and functional hot spots within the protein structure.

## Results

### Model of human ZIP2 based on the previously published human ZIP4 model

**Figure 1:**
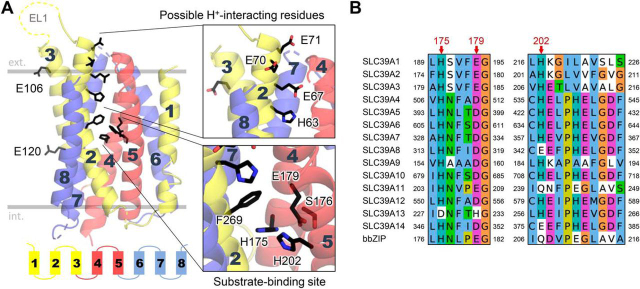
**Structurally and functionally relevant amino acid residues in human ZIP2 (SLC39A2).***A,* model of human ZIP2 based on the previously published model of human ZIP4 (5). Transmembrane helices are numbered *1–8* and colored according to internal structural symmetry. Selected residues that can directly participate in substrate binding are highlighted based on the ZIP4 model. In addition, a cluster of titratable residues on TMH 2 lining the putative pathway to the substrate-binding site is *highlighted*, which might interact with protons. Two acidic residues (Glu-106 and Glu-120) curiously facing the membrane interface are also shown. *B,* conservation of putative Zn^2+^-binding residues on TMHs 4 and 5 are shown in the human SLC39/ZIP family and prokaryotic homolog bbZIP. The complete alignment is available in Fig. S1.

For starting our structure–function studies, we have chosen to construct a homology-based model of human ZIP2 based on a previously published structure of human ZIP4 that was derived from the analysis of co-evolving residue pairs in the protein family (5). The objective of our work was to identify residues within the transmembrane region that could potentially have functional roles in transport. Based on our structural model, we could identify a set of residues that possibly form the substrate-binding site (His-175, His-202, and Glu-179; Fig. 1*A*) based on ZIP4 data (5). These residues seem to be conserved in the human SLC39 family (Fig. 1*B*). Because Zn^2+^ usually binds in a tetrahedral coordination geometry, we were also interested to test whether two nearby residues, Phe-269 and Ser-176, contribute to Zn^2+^ binding during the transport process. A series of titratable residues was also identified on TMH 2 (His-63, Glu-67, Glu-70, and Glu-71; Fig. 1*A*), which we suspected might play a role in substrate recruitment to the binding site, or pH sensitivity of transport previously described by our group (14). Additionally, two acidic residues (Glu-106 and Glu-120) were identified, which extend toward the membrane bilayer (Fig. 1*A*). Their unusual orientation prompted us to add them to the list of residues whose role in transport function we wanted to test.

We next sought to determine the impact of the selected residues on transport activity and pH-dependence of human ZIP2. To this end, selected amino acid residues were substituted by either alanine or, in the case of acidic residues, the protonation-mimicking side-chain glutamine. For His-202, the glutamate substitution was also probed with the anticipation that it might alter transport selectivity, as glutamate is the corresponding residue in human ZIP8 and ZIP14 (Fig. 1*B*), which have been shown to transport Fe^2+^ in addition to Zn^2+^ (27, 28). In the case of Phe-269, which we anticipated might interact with the divalent metal ion substrate via cation–π interactions, the leucine substitution was also tested, because it is a similarly-sized hydrophobic side chain that lacks the aromatic ring and thus is unable to form cation–π interactions.

### Plasma membrane expression and functional activity of ZIP2 single-point variants

**Figure 2:**
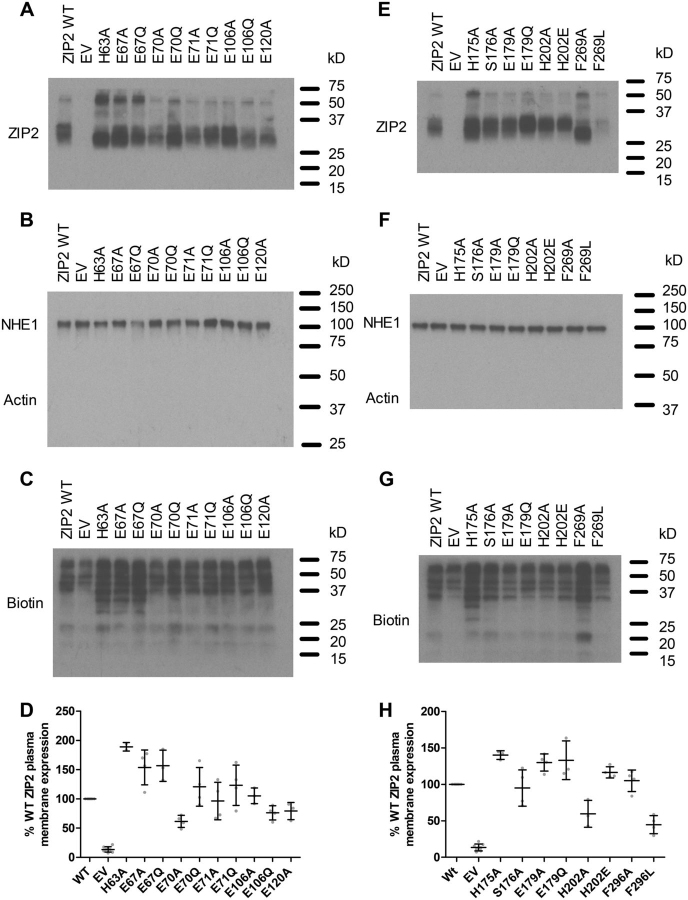
**Plasma membrane expression of generated ZIP2 mutant proteins.** Plasma membranes of transiently transfected HEK293 cells were labeled with sulfo-NHS-LC-biotin and isolated on streptavidin-agarose beads. Representative blots showing the plasma membrane expression of the single-point mutants of the residues present in the proposed H^+^-interaction (*A*) and substrate-binding (*E*) sites. Assessment of the purity of the isolated plasma membranes by the determination of the expression of the Na^+^/H^+^ exchanger (NHE1) and the absence of actin (*B* and *F*) is shown. Determination of biotin labeling of each sample as loading control is shown (*C* and *G*). Relative expression of the indicated proteins was established as the OD of the corresponding bands. Values from three to five independent experiments were normalized to their respective loading controls and expressed as percentage of the expression of WT ZIP2 (*D* and *H*).

The effects of the introduced single-point mutations on the basal plasma membrane expression of human ZIP2 were assessed by immunoblotting membrane protein samples isolated by surface biotinylation. All the generated mutant variants were found to be expressed in the plasma membranes of transiently transfected HEK293 cells; however, some of them showed lower expression levels than WT ZIP2 (Fig. 2, *A* and *E*). Consequently, in all subsequent functional experiments the obtained results for each of the tested mutants were normalized to their respective average plasma membrane surface expression levels (Fig. 2, *D* and *H*).

**Figure 3:**
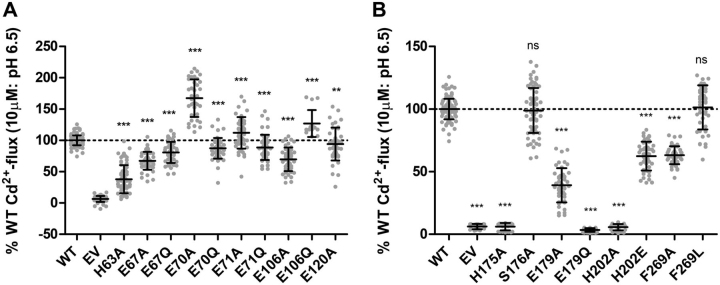
**Functional activity of generated ZIP2 mutant proteins.** Cd^2+^ transport by transiently transfected HEK293 cells expressing the indicated proteins, including the single-point mutants of the residues present on the putative H^+^-interaction (*A*) and substrate-binding sites (*B*). Transport activity was monitored as the change of the fluorescence intensity of the Calcium 5 dye upon perfusion of Cd^2+^ (10 μm) during 10 min (extracellular pH 6.5). The activity was quantified as the AUC and expressed as percentage of the activity determined for WT ZIP2. For each of the indicated proteins, results from three independent experiments (*n* = 32–68) were pooled together and represented as the mean ± S.D. Statistical differences were assessed as described previously (Cd^2+^ uptake by ZIP2 WT *versus* each mutant); *ns*, *p* > 0.05; **, *p* < 0.01; ***, *p* < 0.001.

Next, the functional activities of the generated ZIP2 variants were studied using our fluorescence-based Cd^2+^-flux assay (Fig. 3) (13, 14). All the mutants generated to identify possible H^+^-interaction sites showed altered transport activity (Fig. 3*A*). Remarkably, mutants H63A and E67A and to a lesser extent E106A showed a decrease of ∼60, 35, and 30% in Cd^2+^ transport, respectively, whereas mutants E70A and E106Q showed an increase of ∼65 and 35% in Cd^2+^ transport, respectively. These findings are compatible with a role of these amino acid residues in modulating the effect of extracellular H^+^ on the ZIP2 transport.

With respect to amino acid residues proposed as part of the substrate-binding site (Fig. 3*B*), our results indicate that amino acids His-175, Glu-179, and His-202 might indeed be relevant for substrate binding, but a role for Phe-269 and especially for Ser-176 seems less likely. Mutations of His-175 and His-202 to alanine were completely inactive, but the E179A variant showed a highly significant reduction of Cd^2+^ transport (∼60%). Interestingly, the mutation to glutamine at position 179 completely blocked the transport activity, highlighting the restrictive character of the physicochemical properties required at this position to allow substrate transport. In contrast, conservative mutation of amino acid His-202 to glutamate rescued the activity of ZIP2 by ∼60% if compared with the alanine variant. Mutation to alanine at Ser-176 did not alter transport activity, whereas in the case of F269A variant, the transport activity was reduced by ∼40%. Replacement of Phe-269 by leucine also did not alter transport activity, indicating that the presence of an aromatic residue at that position is not essential for the transport process.

### Substrate transport kinetics

**Figure 4:**
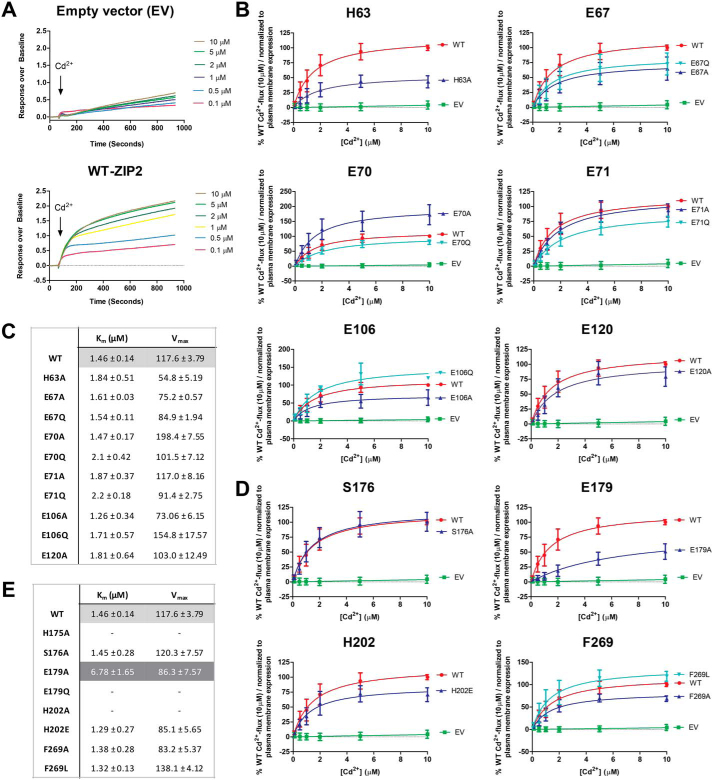
**Cadmium transport kinetics of generated ZIP2 mutant proteins.***A,* representative experiments showing the changes of fluorescence intensity recorded when transiently transfected HEK293 cells expressing ZIP2 WT (*lower panel*) and empty vector (*upper panel*) were perfused with increasing [Cd^2+^] (0. 1–10 μm). Average transport activity by the single-point mutants of the residues present on the proposed H^+^-interaction (*B*) and substrate-binding (*D*) sites in response to increasing [Cd^2+^] at extracellular pH 6.5 (10 min incubation) is shown. Transport activity was quantified as the AUC. For each of the studied proteins, the obtained AUC values were normalized to their respective average plasma membrane expression levels (Fig. 2) and expressed as percentage of the WT activity in response to Cd^2+^ (10 μm). Data from three independent experiments (*n* = 9–20) were pooled together and represented as mean ± S.D. The Michaelis-Menten equation was fit to the represented data (*solid line*). Summary tables of the kinetic parameters calculated for each of the generated mutants related to the putative H^+^-interaction (*C*) or substrate-binding (*E*) sites are shown.

To verify whether the introduced mutations have a direct effect on the binding of the substrate by human ZIP2, kinetic studies of the Cd^2+^ transport (at extracellular pH 6.5) were conducted. ZIP2 showed an apparent affinity for Cd^2+^ of ∼1.46 μm (Fig. 4*A*, *lower panel*), whereas the response of the mock-transfected cells to increasing extracellular [Cd^2+^] lacked Michaelis-Menten kinetics (Fig. 4*A*, *upper panel*). As expected, when compared with ZIP2, the transport kinetics of the mutants generated for the residues proposed as part of the H^+^-interacting sites (Fig. 4*B*) showed differences in the *V*_max_ values but not in the affinity for Cd^2+^, showing *K_m_* values in a similar range to that of WT ZIP2 (Fig. 4*C*). Concerning the mutants of positions predicted as part of the substrate-binding site (Fig. 4*D*), mutants H175A, E179Q, and H202A were not active within the range of tested extracellular [Cd^2+^], supporting the relevance of this cluster of residues for the substrate binding. In contrast, mutants H202E, F269A, and F269L had transport kinetics very similar to those observed for WT ZIP2 (Fig. 4*E*). Interestingly, while still active, the E179A variant displayed a lower apparent affinity for Cd^2+^ of ∼6.78 μm, indicating that the titratable side chain of Glu-179 is directly mediating the binding of the substrate to ZIP2.

### pH-dependence

**Figure 5:**
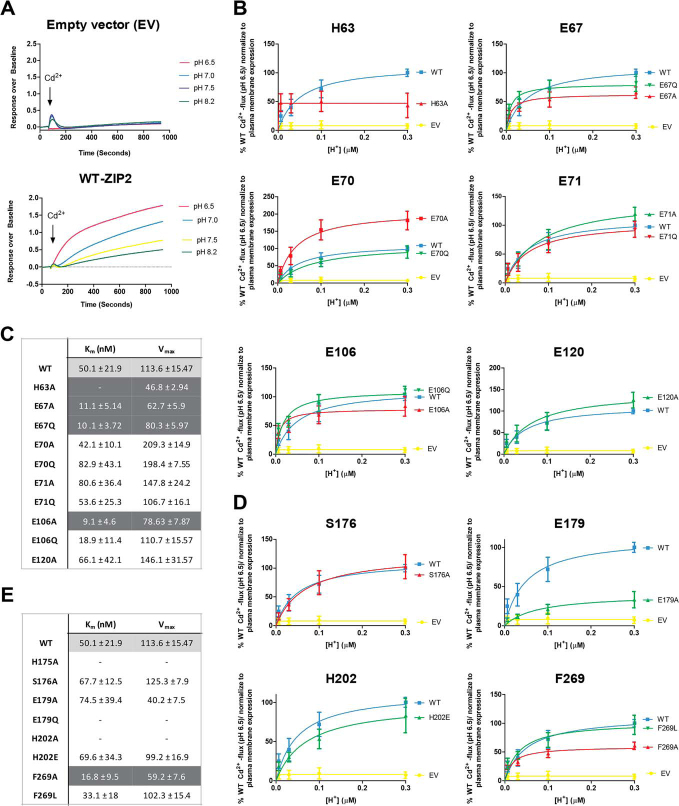
**pH-dependence of Cd^2+^ transport by generated ZIP2 mutant proteins.***A,* representative experiments showing the changes of fluorescence intensity recorded when transiently transfected HEK293 cells expressing ZIP2 WT (*lower panel*) and empty vector (*upper panel*) were perfused with Cd^2+^ (10 μm) at different extracellular [H^+^] (pH 6.5–8.2). Average transport activity by the single-point mutants of the residues present on the proposed H^+^-interaction (*B*) and substrate-binding (*D*) sites in response to Cd^2+^ (10 μm) perfusion in the presence of increasing [H^+^] (10 min) is shown. Transport activity was quantified as the AUC. For each of the studied proteins, the obtained AUC were normalized to their respective average plasma membrane expression levels (Fig. 2) and expressed as percentage of the WT activity in response to Cd^2+^ transport at pH 6.5. Data from three independent experiments (*n* = 8–36) were pooled together and represented as mean ± S.D. The Michaelis-Menten equation was fit to the represented data (*solid line*). Summary tables of the kinetic parameters calculated for each of the generated mutants related to the putative H^+^-interaction (*C*) or substrate-binding (*E*) sites are shown.

To evaluate the effect of extracellular pH on the transport activity of human ZIP2 variants, kinetic studies with a stable substrate concentration (10 μm Cd^2+^) but variable extracellular pH values (6.5–8.2) were conducted. WT ZIP2 showed an apparent affinity for H^+^ of ∼50 nm, corresponding to a pH of ∼7.3 (Fig. 5*A*, *lower panel*). Mock-transfected cells did not exhibit significant Cd^2+^ transport at any of the tested extracellular pH values (Fig. 5*A*, *upper panel*). Of the mutants generated to determine possible H^+^-interacting sites (Fig. 5*B*), variant H63A showed a complete loss of pH-dependence, whereas variants of residues Glu-67 and Glu-106 showed a higher apparent affinity for H^+^ than WT ZIP2, indicating that these residues, together with His-63, are critical for pH-mediated regulation of transport by ZIP2 (Fig. 5*C*). Interestingly, the apparent H^+^ affinity of mutants at residues Glu-70, Glu-71, and Glu-120 were in a similar range to that calculated for WT ZIP2; however, *V*_max_ of these mutants was considerably increased, especially when residues were mutated to alanine (Fig. 5*C*). With respect to the mutants at positions predicted to be part of the substrate-binding site (Fig. 5*D*), as expected, no significant effect on the apparent H^+^ affinity was observed (Fig. 5*E*). However, ZIP2 mutants of residue Phe-269 displayed a higher H^+^ affinity than WT ZIP2 (Fig. 5*E*), suggesting a possible role of this residue in the pH-dependence of the transport process.

### Substrate selectivity

**Figure 6:**
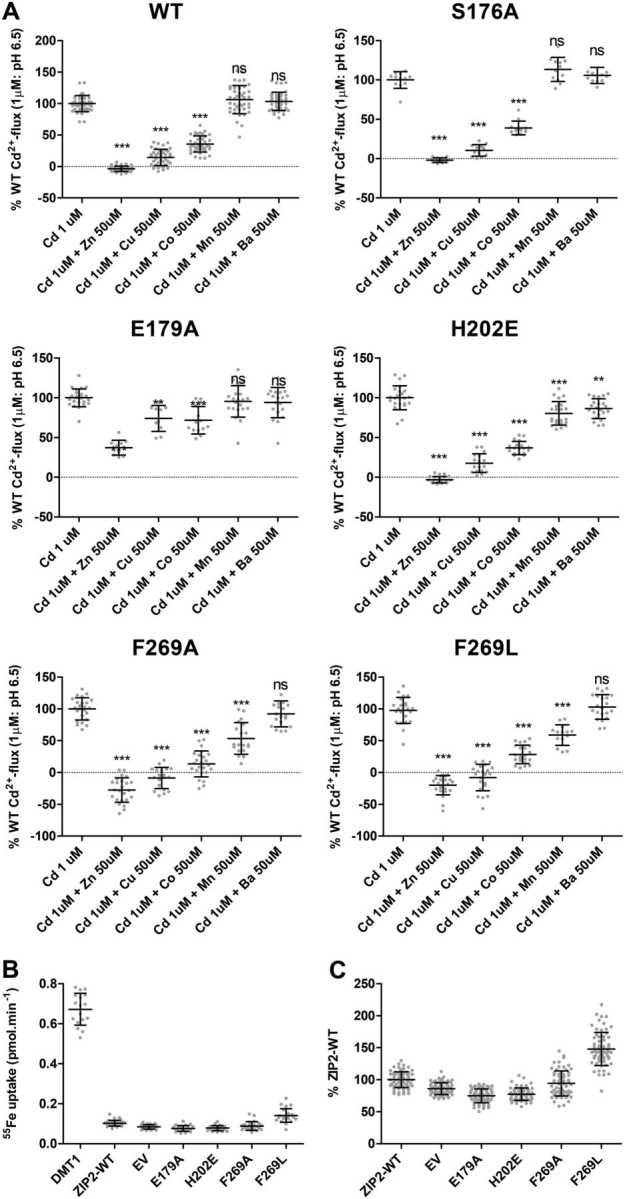
**Apparent substrate selectivity of substrate-binding site–related ZIP2 mutant proteins.***A,* average Cd^2+^ transport by transiently transfected HEK293 cells expressing the indicated proteins. Cd^2+^ (1 μm) uptake was recorded in the presence of an excess (50 μm) of the indicated divalent metals at extracellular pH 6.5 during 10 min. Transport activity was quantified as the AUC and expressed as percentage of the transport in response to Cd^2+^ (1 μm). Data from two to three independent experiments (*n* = 12–24) were pooled together and represented as the mean ± S.D. *B,* representative experiment showing radiolabeled ^55^Fe uptake by transiently transfected HEK293 cells expressing the indicated proteins. ^55^Fe uptake was determined in the presence of 1 μm Fe^2+^ at extracellular pH 5.5 after 15 min of incubation. *C,* average Fe^2+^ transport by transiently transfected HEK293 cells expressing the indicated proteins. For each of the indicated proteins, the ^55^Fe-uptake values were normalized to their respective average plasma membrane expression levels (Fig. 2) and expressed as percentage of the Fe^2+^ transport by WT ZIP2. Data from two independent experiments (*n* = 63–68) were pooled together and represented as mean ± S.D. Statistical differences were assessed as described previously (Cd^2+^ uptake in the absence *versus* the presence of each divalent metal); *ns*, *p* > 0.05; **, *p* < 0.01; ***, *p* < 0.001.

Next, we wanted to elucidate the role of the residues proposed as part of the substrate-binding site (Ser-176, Glu-179, His-202, and Phe-269) in human ZIP2 substrate selectivity (Fig. 6). To this end, Cd^2+^ transport mediated by the functionally active ZIP2 mutants (S176A, E179A, H202E, F269A, and F269L) was measured in the presence of high extracellular concentrations (50 μm) of different divalent metals (Zn^2+^, Cu^2+^, Co^2+^, Mn^2+^, and Ba^2+^). Note that none of these metals induced significant changes on the fluorescent baseline in the absence of Cd^2+^ (data not shown). As described previously by our group for human ZIP2 (14), Mn^2+^ and Ba^2+^ were not able to compete with Cd^2+^ under the tested conditions, whereas the affinity for the other divalent metals decreased as follows: Zn^2+^ > Cu^2+^ > Co^2+^. Mutation to alanine at Ser-176 did not alter the substrate selectivity of ZIP2, which, together with the lack of effect of this mutation on the apparent affinity for Cd^2+^ (Fig. 4*E*), indicates that this residue is not relevant for the substrate binding. Variant E179A showed the same substrate selectivity as WT but with smaller differences between substrates, which, together with its decreased affinity for Cd^2+^ (Fig. 4*E*), indicates an overall reduction of the transport capacity rather than altered substrate selectivity for this variant. Interestingly, variants of residues His-202 and Phe-269 exhibited a broader substrate selectivity, given that Mn^2+^ was able to significantly inhibit Cd^2+^ transport by these variants. Moreover, although the inhibition of Cd^2+^ transport by Mn^2+^ was more pronounced for the Phe-269 variants, H202E-mediated Cd^2+^ transport was also inhibited by Ba^2+^. This might indicate that the size of the side chain at these positions limits the access to the binding site for larger substrates, as in both cases the apparent affinity for Cd^2+^ remained unaltered (Fig. 4*E*). Because the conservative variant H202E is present in other members of the ZIP family (Fig. 1*B*), this might explain why this mutation did not alter the apparent affinity for Cd^2+^ but modified the substrate selectivity. In particular, the H202E variant is present in the ZIP8 and ZIP14 transporters, which have been widely reported to transport iron (28). To test whether Fe^2+^ can be transported by the binding site–related human ZIP2 variants (S176A, E179A, H202E, F269A, and F269L), radiolabeled ^55^Fe-uptake experiments were conducted. Given that WT ZIP2 does not transport Fe^2+^, as a positive control, the human divalent metal transporter SLC11A2/DMT1 was included in the experimental set (Fig. 6*B*). When compared with DMT1-mediated Fe^2+^ uptake, no significant uptake was observed for any of the ZIP2 variants tested. However, among the ZIP2 mutants, F269L seemed to be able to transport Fe^2+^, further supporting the idea that this residue affects the substrate selectivity of human ZIP2, even though it is not directly part of the binding site.

### Voltage-dependence

**Figure 7:**
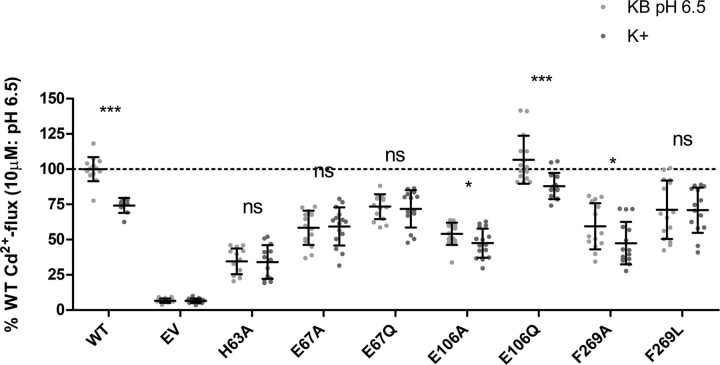
**Voltage-dependence of H^+^-interaction site–related ZIP2 mutant proteins.** Average Cd^2+^ transport by transiently transfected HEK293 cells expressing the indicated proteins in the absence (*light gray dots*) and presence (*dark gray dots*) of high extracellular [K^+^] (140 mm). Cd^2+^ (10 μm) uptake was recorded at extracellular pH 6.5 during 10 min. Transport activity was quantified as the AUC. For each of the indicated proteins, the obtained AUC were normalized to their respective average plasma membrane expression levels (Fig. 2) and expressed as percentage of the transport recorded for ZIP2 WT in standard uptake buffer (KB buffer), pH 6.5. Data from four independent experiments (*n* = 9–15) were pooled together and represented as mean ± S.D. Statistical differences were assessed as described previously (Cd^2+^ uptake in the absence *versus* the presence of 140 mm K^+^); *ns*, *p* > 0.05; *, *p* < 0.05; ***, *p* < 0.001.

As reported previously (14), human ZIP2 transport activity is inhibited in the presence of high extracellular K^+^ as a consequence of the depolarization of the membrane potential due to the K^+^ flux into the cell. Accordingly, we propose that the voltage-dependence described for human ZIP2 could be the consequence of conformational changes due to electrostatic forces exerted by the membrane potential on charged protein residues located within the binding cavity. In this regard, altered electrostatic forces upon membrane depolarization by K^+^ influx could result in structural changes that inhibit the transport process. To test whether this inhibition is related to the residues proposed to be H^+^-interaction sites, the uptake of Cd^2+^ by the human ZIP2 variants showing altered pH-dependence (H63A, E67A, E67Q, E106A, E106Q, F269A, and F269L) (Fig. 5*E*) was determined in the absence and presence of high extracellular K^+^ (Fig. 7). As reported previously, in the presence of high extracellular K^+^, the Cd^2+^ transport mediated by WT ZIP2 is inhibited by ∼25%. Interestingly, a similar effect was observed for mutants E106A, E106Q, and F269A but not for mutants H63A, E67A, E67Q, and F269L. Glu-106 is located in an extracellular loop, whereas Glu-67 and His-63 are located within the binding cavity, in line with our hypothesis that the inhibitory effect induced by depolarization only affects charged residues within the binding cavity. Unexpectedly, at the Phe-269 position, which is located near the binding site, Cd^2+^ transport in the presence of K^+^ upon alanine substitution resembles that of WT ZIP2, whereas when Phe-269 is mutated to leucine, the inhibitory effect is lost.

### Structural model of human ZIP2 based on the prokaryotic bbZIP X-ray structure

**Figure 8:**
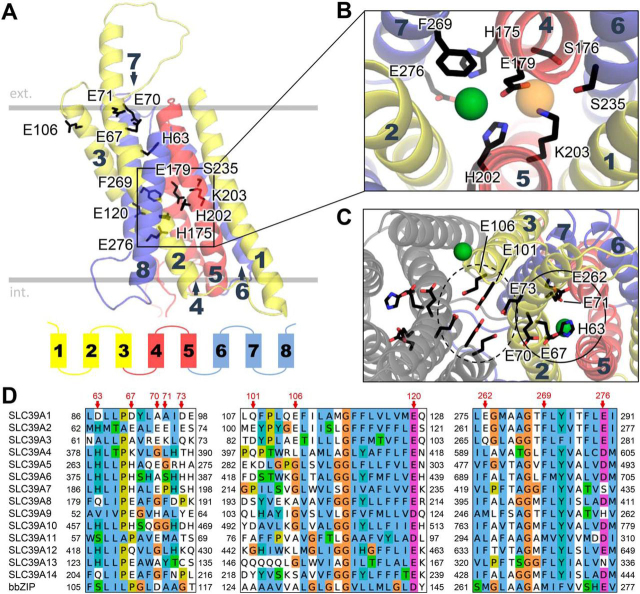
**Model of human ZIP2 based on the bbZIP X-ray structure.***A,* overall structure of human ZIP4 based on the bbZIP X-ray structure (7); the orientation in the bilayer is based on the Orientations of Proteins in Membranes (https://opm.phar.umich.edu/)^7^ (47) prediction for bbZIP. TMHs are numbered *1–8* and colored according to the internal symmetry (TMH 4 is behind and not visible from this angle). Residues tested in this work and shown to be relevant for function are *highlighted. B,* zoom-in on the anticipated substrate-binding site. Position of the bound Zn^2+^ substrate, as anticipated based on the bbZIP X-ray structure, is shown as a *green sphere*. The location of the second bound divalent ion in the bimetal-binding site of bbZIP is shown as a *light orange sphere*. In human ZIP4, the amino group of the Lys-203 side chain occupies this position. In addition, the residue Glu-276 might also participate in substrate binding, as described in the text. *C,* view of the ZIP2 protein from the extracellular side showing two distinct groups of charged/titratable residues, marked by *dashed* and *solid circles*. A putative dimeric arrangement of the proteins is shown (symmetric subunit in *gray*) based on the previously described human ZIP4 model. The location of an additional bound Zn^2+^ ion observed in the bbZIP X-ray structure is shown as a *green sphere* close to Glu-106. *D,* conservation of residues 63–73, 101–120, and 262–276 in the human SLC39/ZIP family and the prokaryotic homolog bbZIP. The complete alignment is available in Fig. S1.

During our work, the structure of a transporter (bbZIP) homologous to the SLC39 family has been solved from *Bordetella bronchiseptica*, at 2.4 Å resolution (7). This protein was crystallized in an inward-open conformation, with a peculiar binuclear metal-binding site, featuring two divalent metal ions. Zhang *et al.* (7) also generated a homology-based model of human ZIP4 (7). However, because many of the residues described by them as part of the substrate-binding site are not conserved in human ZIP2, we decided to generate a structural model of human ZIP2 based on the prokaryotic bbZIP structure. This “new” model of ZIP2 (Fig. 8*A*) is in overall agreement with our previous model based on Antala *et al.* (5) (“old” model, Fig. 1*A*). The residues we have identified as part of the substrate-binding site cluster around the location where the centrally bound Zn^2+^ ion in the bbZIP structure was observed (Fig. 8*B*). Interestingly, the location of the second divalent metal ion observed in the bbZIP structure is occupied by the amino headgroup of the Lys-203 residue in human ZIP4 (Fig. 8*B*). We also observed that due to a rotation of TMH 3 in the “new” structure compared with the “old” structure, the Glu-120 side chain no longer faces the membrane bilayer interface. In addition, the Glu-106 side chain, together with Glu-101 and Glu-73, faces an orientation that could constitute a dimer interface, if the protein was modeled as a dimer based on the dimeric model of human ZIP4 by Antala *et al.* (5) (Fig. 8*C*). These three acidic residues are partially conserved in subfamily II but absent from other members of the SLC39 family (Fig. 8*D*).

Because the hypothesis of a binuclear metal-binding site emerged during our work, we decided to generate additional mutants based on our new model, which we feel is more reliable due to the support of crystallographic data, to investigate the nature of the binding site. In particular, we chose to mutate residues Lys-203 and Ser-235 to test for the presence of the second divalent metal-binding site, as well as Glu-276, which resides in the proximity of the proposed binding site in the new model, and could form the fourth substrate-binding bond, thereby forming a tetrahedral coordination geometry (Fig. 8*B*). For these three residues, alanine substitution as well as conservative (K203R) and uncharged polar (K203Q and E276Q) mutations were generated.

### Plasma membrane expression and functional activity of additional ZIP2 single-point variants

**Figure 9:**
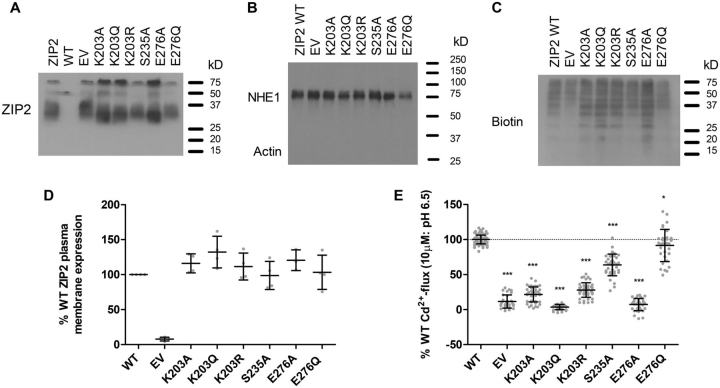
**Plasma membrane expression and functional activity of generated ZIP2 mutant proteins.** Plasma membranes of transiently transfected HEK293 cells were labeled with sulfo-NHS-LC-biotin and isolated on streptavidin-agarose beads. *A,* representative blot showing the plasma membrane expression of the indicated single-point mutant proteins. *B,* assessment of the purity of the isolated plasma membranes by the determination of the expression of the Na^+^/H^+^ exchanger (*NHE1*) and the absence of actin. *C,* determination of biotin labeling of each sample as loading control. *D,* relative expression of the indicated proteins was established as the OD of the corresponding bands. Values from three to five independent experiments were normalized to their respective loading controls and expressed as percentage of the expression of WT ZIP2. *E,* Cd^2+^ transport by transiently transfected HEK293 cells expressing the indicated proteins. Transport activity was monitored as the change of the fluorescence intensity of the Calcium 5 dye upon perfusion of Cd^2+^ (10 μm) during 10 min (extracellular pH 6.5). The activity was quantified as the AUC and expressed as percentage of the activity determined for WT ZIP2. For each of the indicated proteins, results from at least three independent experiments (*n* = 32–44) were pooled together and represented as the mean ± S.D. Statistical differences were assessed as described previously (Cd^2+^ uptake by ZIP2 WT *versus* each mutant); *, *p* < 0.05; ***, *p* < 0.001.

Plasma membrane expression of the generated human ZIP2 mutants was determined by immunoblotting of proteins isolated by surface biotinylation. All the mutants were expressed in the plasma membrane of the transfected cells to a level similar to WT ZIP2 (Fig. 9*A*). Subsequent experiments were normalized to the average plasma membrane expression of each of the mutant proteins (Fig. 9*D*).

Functional activity of the generated mutants was assessed by our fluorescence-based Cd^2+^-flux assay (Fig. 9*E*) (13, 14). Mutants of Lys-203 showed a highly significant reduction on their transport capacity. Cd^2+^ transport by mutants K203Q and K203R was reduced by ∼78 and 72%, respectively, whereas K203Q was completely inactive. This indicates that Lys-203 plays a significant role in the human ZIP2-mediated transport process. Regarding Ser-235, mutation to alanine reduced the transport of Cd^2+^ by ∼36%, suggesting that this residue might also have some functional relevance. Remarkably, mutation to alanine at Glu-276 abolished Cd^2+^ transport completely, whereas mutation to glutamine retained the activity of WT ZIP2 almost completely. This is in line with our hypothesis of Glu-276 being part of the substrate-binding site. These results might also indicate that protonation of Glu-276 is necessary to achieve transport of the substrate, as the E276Q variant behaved like WT ZIP2 and the absence of a side chain at E276A resulted in loss of transport activity.

### Detailed functional characterization of the additional ZIP2 single-point variants

To further evaluate the effect of the mutations on human ZIP2 function, different aspects of the transport mechanism were studied.

**Figure 10:**
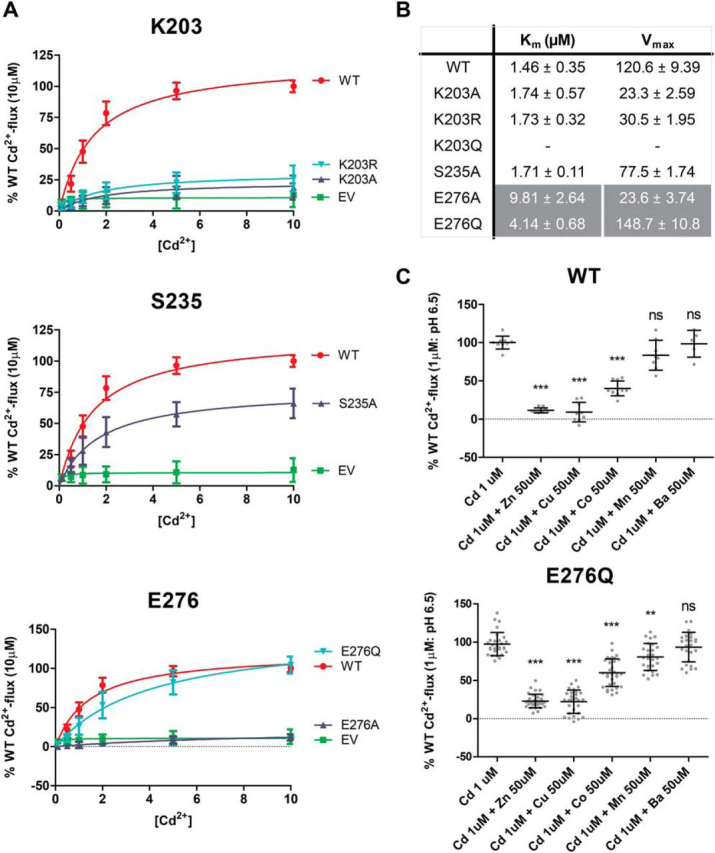
**Cadmium transport kinetics and substrate selectivity of generated ZIP2 mutant proteins.***A,* average transport activity by the single-point mutants in response to increasing [Cd^2+^] (0. 1–10 μm) at extracellular pH 6.5 (10 min incubation). Transport activity was quantified as the AUC. For each of the studied proteins, the obtained AUC values were normalized to their respective average plasma membrane expression levels (Fig. 9) and expressed as percentage of the WT activity in response to Cd^2+^ (10 μm). Data from three independent experiments (*n* = 12–20) were pooled together and represented as mean ± S.D. The Michaelis-Menten equation was fit to the represented data (*solid line*). *B, summary table* of the kinetic parameters calculated for each of the generated mutants. *C,* average Cd^2+^ transport by transiently transfected HEK293 cells expressing the indicated proteins. Cd^2+^ (1 μm) uptake was recorded in the presence of an excess (50 μm) of the indicated divalent metals at extracellular pH 6.5 during 10 min. Transport activity was quantified as the AUC and expressed as percentage of the transport in response to Cd^2+^ (1 μm). Data from two to three independent experiments (*n* = 26–34) were pooled together and represented as mean ± S.D. Statistical differences were assessed as described previously (Cd^2+^ uptake in the absence *versus* the presence of each divalent metal); *ns, p* > 0.05; **, *p* < 0.01; ***, *p* < 0.001.

First, the substrate transport kinetics of the generated mutants were evaluated (Fig. 10). In line with our previous observations, mutations at Lys-203 and Ser-235 exhibited reduced *V*_max_ values compared with WT ZIP2. However, the apparent affinity for Cd^2+^ remained unaltered. This indicates that none of these residues are directly involved in the metal-binding process, hence disproving the hypothesis of a second substrate-binding site. In contrast, mutations at Glu-276 exhibited reduced affinity for Cd^2+^. Calculated *K_m_* values for E276A and E276Q were 7- and 3-fold higher, respectively, than those of WT ZIP. These findings confirm our hypothesis of Glu-276 being the missing metal-coordinating point for the proposed binding site. Accordingly, we decided to assess the impact of this mutation on the substrate selectivity of the transporter (Fig. 10*C*). Substrate selectivity remained similar to WT; however, the overall Cd^2+^ transport activity in the presence of Zn^2+^, Cu^2+^, and Co^2+^ was ∼2, ∼2, and ∼1.5-fold higher than WT, respectively. This result, together with the significant inhibition of Cd^2+^ transport by Mn^2+^ by ∼20%, further supports the role of Glu-276 as part of the substrate-binding site. The overall decreased inhibition of Cd^2+^ transport by the other metals might reflect the lower substrate affinity of this variant, although its capacity to transport Mn^2+^ in contrast to WT ZIP2, even if minimal, indicates that this residue might also be relevant to the selectivity of the transporter.

**Figure 11:**
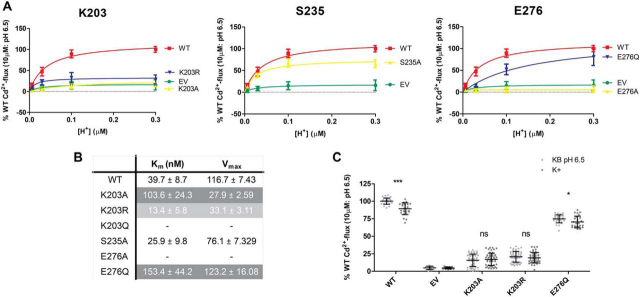
**pH-dependence of Cd^2+^ transport by generated ZIP2 mutant proteins.***A,* average transport activity by the single-point mutants in response to Cd^2+^ (10 μm) perfusion in the presence of increasing [H^+^] (pH 6.5–8.2) (10 min). Transport activity was quantified as the AUC. For each of the studied proteins, the obtained AUC were normalized to their respective average plasma membrane expression levels (Fig. 9) and expressed as percentage of the WT activity in response to Cd^2+^ transport at pH 6.5. Data from three independent experiments (*n* = 8–28) were pooled together and represented as mean ± S.D. The Michaelis-Menten equation was fit to the represented data (*solid line*). *B, summary table* of the kinetic parameters calculated for each of the generated mutants. *C,* average Cd^2+^ transport by transiently transfected HEK293 cells expressing the indicated proteins in the absence (*light gray dots*) and presence (*dark gray dots*) of high extracellular [K^+^] (140 mm). Cd^2+^ (10 μm) uptake was recorded at extracellular pH 6.5 during 10 min. Transport activity was quantified as the AUC. For each of the indicated proteins, the obtained AUC were normalized to their respective average plasma membrane expression levels (Fig. 9) and expressed as percentage of the transport recorded for ZIP2 WT in standard uptake buffer (KB buffer), pH 6.5. Data from four independent experiments (*n* = 25–53) were pooled together and represented as mean ± S.D. Statistical differences were assessed as described previously (Cd^2+^ uptake in the absence *versus* the presence of 140 mm K^+^); *ns, p* > 0.05; *, *p* < 0.05; ***, *p* < 0.001.

Next, the effect of the extracellular pH on the transport mediated by the generated mutants was assessed (Fig. 11). Interestingly, except for Ser-235, all the mutants showed altered apparent affinity for H^+^. Nevertheless, although for mutants K203A and E276Q the apparent affinity for H^+^ was reduced by ∼2.5- and 4-fold, respectively, and for mutant K203R it was increased by ∼3-fold. These results suggest that even though the Lys-203 is not directly involved in metal binding, its protonation state seems to be highly relevant to the electrostatic balance of the substrate-binding cavity. Likewise, Glu-276 is also involved in the pH sensitivity of the transport process, as the mutation to a “protonated-like” form (E276Q) substantially altered the pH-dependence of the transport process. To further study the contribution of these residues to the electrostatic balance of the binding cavity, the voltage-dependence of the mutants were studied (Fig. 11*C*). Interestingly, mutations at Lys-203 abolished the effect of K^+^ on Cd^2+^ transport, confirming the contribution of this residue to the electrostatic balance and overall voltage-dependence of the transporter. In contrast, the E276Q mutant was still slightly inhibited by K^+^, which indicates that its contribution to this aspect of the transport process is limited.

### pK*_a_* calculations

Because we have observed a cluster of charged residues lining TMH 2, some of which also seem to determine the pH sensitivity of transport and apparent binding affinity toward protons (His-63 and Glu-67), we suspected that perturbation of these side chains by mutation would affect the local electrostatic environment, and thus the binding affinity for protons. Therefore, we performed calculations to estimate the p*K_a_* value of titratable protein side chains in ZIP2, also taking into account the membrane environment. First, we tested the effect of the presence of the Zn^2+^ substrate in the proposed binding site (Fig. 8*B*), which caused a marked shift in side chain p*K_a_* values of directly coordinating residues, *i.e.* Glu-179, His-175, and His-202 (Table 1). A marked shift was also observed for Glu-276 and Lys-203. Interestingly, the p*K_a_* of Lys-203 due to the presence of the substrate dropped to physiological values (6.69). A slight shift due to the presence of the substrate was also observed for residues in the charged cluster near His-63 (including His-63, Glu-67, Glu-71, and Glu-262). As a second step, we repeated the calculations with mutant variants of our human ZIP2 model. The introduction of the H63A substitution, which completely abrogated pH-dependence of transport in our experiments, only affected the p*K_a_* of Glu-262 with a downward shift of more than 0.1 unit (Table 1 and Table S1). Interestingly, introduction of either K203A or K203Q mutation caused a dramatic shift of the side-chain p*K_a_* of Glu-179, raising it closer to physiological values (∼7.6–8.0). In the K203R variant, the Arg-203 side chain was predicted to have a p*K_a_* 2.12 units higher than Lys-203 in WT, whereas the p*K_a_* values of all other residues were shifted by less than 1 unit (Table 1 and Table S1).

**Table 1:** Estimated p*K_a_* values of selected side chains of ZIP2 Values have been calculated both in the presence and absence of a Zn^2+^ ion substrate, in wildtype protein, and upon H63A, K203A, and K203R substitutions. Δp*K_a_* shifts denote differences between apo and Zn^2+^-bound p*K_a_* values. Residues with shaded background were protonated during the calculations. Values marked with an asterisk correspond to the Arg-203 side chain of the K203R variant. The full list of p*K_a_* values for all side chains can be found in Table S1.

## Discussion

Our combined *in silico* and *in vitro* efforts successfully identified key residues that are responsible for substrate binding and pH sensitivity of human ZIP2. In addition, based on structural comparison of our old and new models of ZIP2, we present a hypothetical mechanism of metal ion transport.

### Substrate-binding site

Based on our results, it seems evident that Glu-179, His-175, His-202, and Glu-276 directly bind the divalent metal ion substrate, which is in line with previous studies on SLC39 proteins (5), as well as our modeling efforts (Figs. 1*A* and 8, *A* and *B*). Importantly, our model of ZIP2 based on the prokaryotic bbZIP structure suggests that the binding site of human ZIP2 is only able to bind a single divalent ion. Indeed, our experiments suggest that removal of the nonconserved side chains forming the second binding site in bbZIP (Lys-203, Ser-176, and Ser-235) does not affect the binding affinity of the substrate. Instead, we hypothesize that the presence of a positive charge in the form of a proton near the analogous residues of the second metal ion–binding site in bbZIP is essential for transport in human ZIP2. In the WT protein, this charge is represented by the proton carried by the side chain of Lys-203, which also coordinates the side chain of Glu-179 to an optimal substrate-binding orientation. In the K203A variant, the proton might bind to the Glu-179 side chain instead. The predicted p*K_a_* of Glu-179 in the K203A mutant (7.98, Table 1) is lower than that of Lys-203 in the WT protein (10.31), which is in line with the observed downward shift in proton-binding affinity of K203A, compared with WT. In case of the K203R variant, the estimated p*K_a_* of Arg-203 is higher than WT (12.43), corresponding to the higher observed proton-binding affinity of this variant. However, it is likely that the larger Arg residue fails to efficiently coordinate the Glu-179 residue in a competent substrate-binding orientation, or it causes other structural distortions that severely impact the function of the protein. In case of the K203Q variant, which proved to be nonfunctional in our experiments, the introduced Gln residue might form H-bond interactions with the nearby Glu-179 side chain, thus preventing proton binding and rendering the transporter nonfunctional. The single divalent binding site seems to be a unique property of subfamily II of the SLC39 family (SLC39A1–3) based on sequence conservation, as opposed to members of the LIV-1 subfamily (SLC39A4–8, SLC39A10, and SLC39A12–14 (4)) as well as bbZIP. In addition, although likely not directly participating in substrate binding, the nearby residue Phe-269 seems to affect substrate selectivity, possibly by regulating the size of the access path to the substrate-binding site. When comparing the two models of human ZIP2 presented here, even though they are very similar overall, we believe that the bbZIP-based model recapitulates the geometry of the proposed binding site better than the apo old model, because it presumes that there is already a bound divalent metal ion substrate. Based on our experimental findings and the underlying crystallographic data, we believe that our new model represents the inward-open state of human ZIP2 more precisely and reliably.

### Residues shaping the pH sensitivity of transport

Apart from the residues near the proposed substrate-binding site, we have identified several other residues that affect the pH sensitivity of transport in ZIP2 as well as its apparent affinity for protons. The most prominent one is His-63, the loss of which renders the transporter completely pH-insensitive. In addition, mutations of Glu-67 and Phe-269, the two residues tested that are closest to His-63, also affect apparent proton affinity and thus pH sensitivity of transport (Fig. 5*C*). We therefore suspect that His-63 and its vicinity (including Glu-67 and Phe-269) represent a functional hot spot within the protein structure. However, based on our structural model and p*K_a_* estimations, His-63 is probably not protonated in the inward-open state of human ZIP2. Based on its location, we propose that His-63 is part of an extracellular gate, the closing probability of which might be enhanced by elevated H^+^ concentration. In general, we suggest that transport by ZIP2 is very sensitive to local electrostatic perturbations, such as in the case of membrane depolarization or protonation changes. Our p*K_a_* estimations show that there could be electrostatic cross-talk between the extracellular gate and the substrate-binding site, whereas mutations of charged residues around the substrate-binding site (Lys-203 and Glu-276) alter pH sensitivity and transport efficiency. We also suggest that Glu-70, Glu-71, and Glu-120 are constantly protonated, based on p*K_a_* estimation and the fact that their glutamine-substituted variants display WT-like pH sensitivity. Interestingly, mutations of Glu-106 also affected apparent proton affinity and pH sensitivity of transport. A hypothetical dimeric structure suggests that Glu-73, Glu-101, and Glu-106 might form a cluster of acidic residues with a possible functional role. We propose that this site might be a sensor for extracellular proton concentration, or possibly a lipid-binding site, exerting its functional role by the allosteric stabilization of the extracellular gate (Fig. 8*C*). However, because neither Glu-73 nor Glu-101 were tested in our study, this hypothesis warrants further investigation.

### Possible transport mechanism based on internal symmetry considerations

The structural fold of ZIP transporters bears the “3 + 2 + 3” TMH organization and shows an internal structural symmetry (4), which potentially provides a structural basis for an alternating-access transport mechanism (29). We believe that such a mechanism would be better supported, compared with a channel-like mechanism, by the following facts: 1) substrate transport through ZIPs is saturable; 2) the bbZIP structure is closed on the extracellular side with no visible pore-like structures; and 3) a well-defined substrate-binding site is present. Although the bbZIP protein was crystallized in an inward-open conformation, the model of human ZIP4 by Antala *et al.* (5) seems to be in a substrate-free occluded state, with both extracellular and intracellular gates closed. The latter could be an artificial state due to model construction (30) and might be less physiologically relevant. Interestingly, it exhibits a conformation with features of both inward-facing and outward-facing states. For this reason, comparison of the models of human ZIP2 based on these two structures can be a valuable source of inspiration to derive hypotheses about possible transport mechanisms. Notably, His-63 lies in a bulge along TMH 2, which is likely present due to the helix-breaking residue Pro-110 in bbZIP. Such regions can represent hinge points in the protein chain, suggesting that this region around His-63, Glu-67, and Glu-262 might undergo rearrangements during the opening of the extracellular gate. Consistent with this, the residue equivalent to His-63 in the other symmetric half of the transporter (given the internal structural symmetry) is Glu-276. According to our observations, this residue directly coordinates the metal ion substrate. Thus, Glu-276 might constitute an intracellular gate, the dislocation of which could disrupt the substrate-binding site and enable the solvation of the bound substrate ion. Residues downstream of Glu-276 also include a helix-breaking residue (Pro-279) and are probably disordered in the inward-facing state, as their coordinates could not be resolved in the bbZIP structure. A comparison of our two ZIP2 models also shows that the intracellular half of TMH 4 is significantly more open in the bbZIP-based model, indicating that this region could be part of the intracellular gate as well. The apparent hinge point lies around His-175 in human ZIP2, which binds one of the five additional Zn^2+^ ions in the bbZIP structure, along with the residue corresponding to Glu-276. Based on our old ZIP2 model, we propose that, in a substrate-bound inward-closed state, both His-175 and Glu-276 bind the central Zn^2+^ substrate and that intracellular closure is achieved by the α-helical reorganization of residues 277–283 and the closure of the intracellular parts of TMH 4 and TMH 7. In turn, based on the symmetry considerations, the extracellular half of TMH 5 positioned *vis à vis* His-63 is expected to form part of the extracellular gate as the symmetry pair of TMH 4. In this case, His-202 would constitute a hinge point, where a marked kink in TMH 5 is already evident. The residue corresponding to His-63 in bbZIP (Ser-106) was suggested to play a role in guiding metal substrate into the transport pathway (7), whereas alanine substitution of His-379, the corresponding residue in human ZIP4, severely reduced transport activity (5). These considerations underline our hypothesis that stabilization of the region around His-63 (including Glu-67 and Glu-262) via protonation could contribute to the closure of the extracellular gate, constituting a possible mechanism of how proton concentration could affect the transport rate. In addition, the integrity of the hot spot around His-63 seems to be important for voltage-modulated transport via human ZIP2, because mutations in this region (H63A, E67A/E67Q, and F269L) lose their sensitivity to depolarizing conditions (Fig. 7). This might also indicate that charged residues in the region (*e.g.* Glu-67 or possibly also Glu-262) are mobile and that their rearrangement upon changes in membrane potential directly affects the rate of transport. Therefore, based on our experimental evidence, we propose that the stability of this functional hot spot is a key aspect in the transport cycle, and thus, the elements that can influence the arrangement of this particular region, as protonation state or charge distribution, are able to influence the transport rate through ZIP2. Further studies will be required to clarify the exact role of these hot spot residues in the overall transport mechanism of ZIP2.

## Experimental procedures

### Materials

All chemicals and reagents were purchased from Sigma except when mentioned specifically.

### Human ZIP2 single-point mutant generation

Amino acid residues present in His-63, Glu-67, Glu-70, Glu-71, Glu-106, Glu-120, His-175, Ser-176, Glu-179, His-202, and Phe-269 as well as Lys-203, Ser-235, and Glu-276 were mutated by PCR amplification of the human WT pIRES2-DsRed-ZIP2 construct (13) using primers (sequences available upon request) designed to replace these residues as follows: Glu to Ala or Gln; His to Ala, Gln, or Glu; Lys to Ala, Gln, or Arg; Ser to Ala; and Phe to Ala or Leu. Obtained PCR products were used to transform XL1-Blue MRF super-competent cells (Agilent Technologies) by the heat-shock method. Transformed cells were selected in LB-agar plates containing 30 μg/ml kanamycin. DNA from single-clone colonies was isolated and sequenced (Microsynth AG) to verify the proper insertion of the designed mutations.

### Cell culture

HEK293 were obtained from American Type Culture Collection (ATCC, Manassas, VA). HEK293 cells were cultured in Dulbecco’s modified Eagle’s medium (DMEM) (Invitrogen) supplemented with 10% fetal bovine serum, 10 mm HEPES, 1 mm sodium pyruvate (Gibco), and 0.1 mm nonessential amino acids in a cell culture incubator under standard conditions for mammalian cells (37 °C; 5% CO_2_).

### Transient transfection

HEK293 cells were plated on poly-d-lysine–coated 6 wells (2 ml of 500,000 cells/ml solution per well) or 96 wells (100 μl of 300,000 cells/ml solution per well). After 24 h, cells were transfected with the desired DNA construct and Lipofectamine 2000 (Invitrogen), following the instruction described in the manufacturer’s protocol. 6 h after the transfection, the medium was completely replaced by fresh DMEM. The subsequent experiments were performed 24 h after the transfection.

### Cell-surface biotinylation and Western blotting

Transiently transfected HEK293 cells (6-well plates) were rinsed with PBS and incubated with 1.5 mg/ml sulfo-NHS-SS-biotin for 1 h at 4 °C. Next, cells were washed with PBS buffer supplemented with 1 mm MgCl_2_, 0.1 mm CaCl_2_, and 100 mm glycine and then newly rinsed with PBS. Finally, cells were lysed with radioimmunoprecipitation assay buffer (RIPA) (150 mm NaCl, 5 mm EDTA, 1% Triton X-100, 0.5% deoxycholate, 0.1% SDS, 50 mm Tris-HCl, pH 7.4) containing fresh protease inhibitor mixture (Roche Applied Science). Cell lysates were equilibrated overnight at 4 °C with streptavidin-agarose beads equivalent to the protein content of the lysates. Next day, beads were washed and recovered by centrifugation. Biotinylated proteins were released from the beads by heating to 95 °C with 2× Laemmli buffer, separated on SDS-polyacrylamide gels, and transferred onto Immobilion-P membrane blots (Millipore). Blots were incubated sequentially with primary and secondary antibodies, and proteins were revealed by enhanced chemiluminescence method (ECL). Primary antibodies used were as follows: rabbit polyclonal anti-SLC39A2 antibody (1:500 dilution) (Abcam); mouse monoclonal anti-Na^+^/H^+^ exchanger 1 antibody (1:1000) (Merck Millipore); and mouse monoclonal anti-β-actin (1:100 dilution) (Santa Cruz Biotechnology). Secondary antibodies used were as follows: HRP-conjugated goat anti-mouse IgG (1:3000) (Bio-Rad) and goat anti-rabbit IgG (1:20,000) (Promega). To verify the equal loading among samples, all the biotinylated proteins were visualized with avidin–HRP conjugate (1:1000 dilution) (Bio-Rad). To assess the expression of each of the transfected proteins, densitometry determination of the visualized bands was performed using the ImageJ software (National Institutes of Health) (31).

### Intracellular cadmium accumulation measurements by real-time fluorescence imaging

After removing the culture medium, transient transfected HEK293 cells (poly-d-lysine–coated 96-well, clear bottom, black plates) were loaded with 50 μl of calcium 5 fluorescent dye (Molecular Devices) dissolved in uptake buffer (117 mm NaCl, 4.8 mm KCl, 1 mm MgCl_2_, 10 mm glucose, 5 mm HEPES, 5 mm MES, pH 7.4) at 37 °C for 1 h. Next, Cd^2+^ accumulation was determined by quantifying the fluorescence signal (excitation, 470–495 nm; emission, 515–575 nm), a consequence of the binding of internalized Cd^2+^ to the intracellular Calcium 5 dye using a FLIPR Tetra microplate reader (Molecular Devices). In each experiment, a baseline was recorded for 50 s, and then, 50 μl of uptake buffer containing 2× [Cd^2+^] (0.1–10 μm) were added, and changes in fluorescence intensity were recorded for 15 min. To quantify the transport activity mediated by the transfected ZIP2 variants, the area under the curve of the fluorescence signal recorded between the Cd^2+^ perfusion step and the end of the experiments was calculated. For the pH-dependence experiments, pH levels were adjusted with 1 n HCl/NaOH. For the metal competition experiments, high concentrations (50 μm) of the tested divalent metals (Zn^2+^, Cu^2+^, Co^2+^, Mn^2+^, and Ba^2+^) were perfused together with Cd^2+^ (1 μm). When studying the effect of [K^+^] on the transport activity of ZIP2 variants, in the uptake buffer [NaCl] was replaced by equimolar [KCl].

### Radiolabeled iron uptake

These experiments were conducted as described previously (32). Briefly, media were removed from the culture plates (poly-d-lysine–coated 96-well, clear bottom, white plates), and the cells were rinsed three times with uptake buffer (140 mm NaCl, 2.5 mm KCl, 1 mm CaCl_2_, 1 mm MgCl_2_, 1.2 mm K_2_HPO_4_, 100 mm glucose, 5 mm HEPES, 5 mm MES, pH 7.4). Then, 100 μl of uptake buffer containing 1 μm nonradioactive FeCl_2_, 1 mm ascorbic acid, and 0.5 μCi/ml radioactive ^55^Fe (American Radiolabeled Chemicals, St. Louis, MO) were added into each well. Cells were incubated at room temperature for 15 min, and the uptake was stopped by washing the plates three times with ice-cold uptake buffer. Finally, 100 μl of Microscint 20 (PerkinElmer Life Sciences) were added to each well, and the uptake activity was determined as counts/min using a TopCount Microplate Scintillation Counter (PerkinElmer Life Sciences).

### Structural modeling and sequence alignments

The alignment of the human SLC39/ZIP family and bbZIP was generated using PSI-COFFEE 11.00.d625267 using default settings on the server http://tcoffee.crg.cat/7 (33, 34). The complete alignment can be found in Fig. S1. The Uniprot sequence Q9NP94 (isoform 1) was used for modeling human ZIP2. The first ZIP2 model was generated based on the published structural model of human ZIP4 by Antala *et al.* (5), using Rosetta (35) version 2016.20.58704. First, a sequence alignment between the ZIP4 model and ZIP2 was generated using ClustalW (36), and then the human ZIP2 sequence was threaded 100 times on the human ZIP4 model using the “fixbb” program of Rosetta with default settings. The model with the best Rosetta score (“talaris2014” scoring function) was selected and optimized using the “fast_relax” protocol with “repeats” set to 8 using the “relax” tool of Rosetta (37), in 10 independent runs. In this step, transmembrane segments as predicted by the MESSA consensus server (38) were taken into account, and the “mpframework_smooth_fa_2012” scoring function (39) was used to mimic the membrane environment around the protein. In the above-mentioned protocol, dimeric symmetry restraints were applied throughout. Finally, the model with the best score was selected. For modeling the structure of human ZIP2 based on bbZIP, MODELLER 9.17 (40) was used with default settings, and an alignment between the sequence of the bbZIP structure (Protein Data Bank code 5TSA (7)) and human ZIP2 was generated using AlignMe (41). Secondary structure restraints were added to keep residues 24–33 and 67–83 in an α-helical conformation based on secondary structure prediction by the PSIPRED 3.3 server (42, 43). 50 models were generated, and the model with the lowest MODELLER objective function score was selected.

### pK*_a_* calculations

The p*K_a_* values of protein residue side chains were estimated using PROPKA 3.1 (44, 45), followed by the calculation of p*K_a_* shifts due to the membrane bilayer using APBSmem (45). The initial protonation states for all titratable residues were chosen based on PROPKA 3.1 predictions alone. Then, APBSmem calculations were performed iteratively by adjusting the protonation state of residues after each iteration based on the predicted p*K_a_* values until reaching self-consistency. In the iterative scheme, residues with a predicted p*K_a_* higher than 6.5 were protonated (corresponding to a pH of 6.5). For PROPKA 3.1, default options were used. For APBSmem, the PARSE force-field was used, 0.1 m monovalent counterions with 2.0 Å radius, 298.15 K temperature, and the dielectric constants of protein, solvent, membrane, and headgroup were set to 10.0, 80.0, 2.0, and 80.0, respectively. Membrane and headgroup thickness of 42.0 and 9.0 Å were used, respectively, with the membrane bottom at −21.0 Å. Calculations were performed with the *npbe* method, *Spl2* charge model, and three-step grid refinement as described elsewhere (46).

### Statistics

Normal distribution of the collected data groups was assessed by Kolmogorov-Smirnov and Shapiro-Wilk tests (*n* < 50 samples). Normally distributed sample groups were compared using unpaired Student’s test, and the nonparametric data groups were compared using the Mann-Whitney *U* test. Statistical tests were performed using the IBM SPSS statistics 20 software. Significance level was set at *p* < 0.05.

## Author contributions

G. G., G. A., M. A. H., and J. P.-G. conceptualization; G. G., G. A., D. G. F., and J. P.-G. data curation; G. G. software; G. G., G. A., D. G. F., and J. P.-G. formal analysis; G. G., D. G. F., M. A. H., and J. P.-G. supervision; G. G., G. A., D. G. F., M. A. H., and J. P.-G. validation; G. G., G. A., D. G. F., and J. P.-G. investigation; G. G., G. A., D. G. F., M. A. H., and J. P.-G. visualization; G. G., G. A., D. G. F., and J. P.-G. methodology; G. G., G. A., D. G. F., M. A. H., and J. P.-G. writing-original draft; G. G., G. A., D. G. F., M. A. H., and J. P.-G. writing-review and editing; D. G. F., M. A. H., and J. P.-G. resources; D. G. F. and M. A. H. funding acquisition; M. A. H. and J. P.-G. project administration.

## Footnotes

6 The abbreviations used are:

TMH: transmembrane helix
bbZIP: *B. bronchiseptica* ZrT/Irt-like protein
HRP: horseradish peroxidase
DMEM: Dulbecco's modified Eagle's medium
AUC: area under the curve.

7 Please note that the JBC is not responsible for the long-term archiving and maintenance of this site or any other third party hosted site.
