# Supplementary material for: Unraveling the structural elements of pH sensitivity and substrate binding in the human zinc transporter SLC39A2 (ZIP2)
Source: J Biol Chem. 2020 Aug 26;294(20):8046–63. doi: 10.1074/jbc.RA118.006113 (PMC6527156; doi:10.1074/jbc.RA118.006113)
Supplement: Supplementary file 1 [file mmc1.docx]

Supplementary Information

Unraveling the structural elements of pH sensitivity and substrate binding in the human zinc transporter SLC39A2 (ZIP2)

**^1, 3*^ Gergely Gyimesi, ^1, 2, 3*^ Giuseppe Albano, ^1, 2, 3^ Daniel G. Fuster, ^1, 2, 3, **, #^ Matthias A. Hediger and ^1, 2, 3#^ Jonai Pujol-Giménez**

From ^1^University of Bern, Institute of Biochemistry and Molecular Medicine, Bühlstrasse 28, 3012 Bern, Switzerland; ^2^National Center of Competence in Research, NCCR TransCure, Bühlstrasse 28, 3012 Bern, Switzerland; ^3^Department of Nephrology and Hypertension, Inselspital, Bern University Hospital, University of Bern, Freiburgstrasse 18, 3010 Bern, Switzerland

Running title: Structural basis of transport in SLC39A2

***** Both authors have contributed equally to the present work

^**^ Co-corresponding author

^#^ To whom correspondence should be addressed:

Jonai Pujol-Giménez: Department of Nephrology and Hypertension, Inselspital, Bern University Hospital, University of Bern, Freiburgstrasse 18, 3010 Bern, Switzerland
E-mail: [jonai.pujol@dbmr.unibe.ch](mailto:jonai.pujol@dbmr.unibe.ch) Tel. +41 31 632 94 76

Or

Matthias A. Hediger: Same address as above. E-mail: [matthias.hediger@ibmm.unibe.ch](mailto:matthias.hediger@ibmm.unibe.ch)

**Supplementary Figure 1.**

Alignment of the human SLC39/ZIP family and the prokaryotic *Bordetella bronchiseptica* homologue, bbZIP. Corresponding Uniprot accession numbers are given in parentheses.

**Supplementary Table 1.**

Estimated p*K_a_* values of titratable side-chains of hZIP2. Values have been calculated both in the presence and absence of a Zn^2+^ ion substrate and upon H63A, K203A, K203Q and K203R substitutions. Residues with shaded background were protonated during the calculations. Values marked with an asterisk (*) correspond to the R203 side-chain of the K203R variant.

|  |  | **WT apo** | **WT Zn^2+^** | **H63A apo** | **H63A Zn^2+^** | **K203A apo** | **K203A Zn^2+^** | **K203Q apo** | **K203Q Zn^2+^** | **K203R apo** | **K203R Zn^2+^** |
| --- | --- | --- | --- | --- | --- | --- | --- | --- | --- | --- | --- |
| ASP | 35 | **3.88** | **3.86** | **3.88** | **3.86** | **3.88** | **3.87** | **3.88** | **3.87** | **3.88** | **3.86** |
| ASP | 94 | **3.46** | **3.44** | **3.44** | **3.42** | **3.46** | **3.46** | **3.46** | **3.46** | **3.46** | **3.44** |
| ASP | 96 | **5.79** | **5.72** | **5.74** | **5.68** | **5.79** | **5.77** | **5.79** | **5.77** | **5.79** | **5.72** |
| ASP | 138 | **3.96** | **3.95** | **3.96** | **3.95** | **3.96** | **3.95** | **3.96** | **3.95** | **3.96** | **3.95** |
| ASP | 249 | **18.79** | **18.40** | **18.83** | **18.46** | **18.77** | **18.69** | **18.74** | **18.66** | **18.76** | **18.37** |
| GLU | 2 | **4.61** | **4.57** | **4.61** | **4.57** | **4.62** | **4.61** | **4.61** | **4.60** | **4.60** | **4.56** |
| GLU | 67 | **5.87** | **5.75** | **5.82** | **5.68** | **5.88** | **5.81** | **5.87** | **5.81** | **5.87** | **5.75** |
| GLU | 70 | **6.93** | **6.90** | **6.89** | **6.85** | **6.93** | **6.92** | **6.93** | **6.91** | **6.93** | **6.90** |
| GLU | 71 | **11.10** | **10.67** | **11.07** | **10.67** | **11.09** | **10.92** | **11.06** | **10.88** | **11.08** | **10.64** |
| GLU | 73 | **5.70** | **5.68** | **5.70** | **5.68** | **5.70** | **5.69** | **5.70** | **5.69** | **5.70** | **5.68** |
| GLU | 88 | **4.56** | **4.56** | **4.56** | **4.56** | **4.56** | **4.56** | **4.56** | **4.56** | **4.56** | **4.56** |
| GLU | 101 | **4.84** | **4.83** | **4.84** | **4.83** | **4.84** | **4.84** | **4.84** | **4.83** | **4.84** | **4.83** |
| GLU | 106 | **13.64** | **13.55** | **13.66** | **13.56** | **13.64** | **13.58** | **13.64** | **13.58** | **13.65** | **13.55** |
| GLU | 120 | **8.96** | **8.27** | **9.01** | **8.33** | **8.87** | **8.67** | **8.83** | **8.63** | **8.94** | **8.23** |
| GLU | 139 | **4.55** | **4.55** | **4.55** | **4.55** | **4.55** | **4.55** | **4.55** | **4.55** | **4.55** | **4.55** |
| GLU | 140 | **4.50** | **4.50** | **4.50** | **4.50** | **4.50** | **4.50** | **4.50** | **4.50** | **4.50** | **4.50** |
| GLU | 148 | **4.55** | **4.53** | **4.55** | **4.53** | **4.55** | **4.54** | **4.55** | **4.54** | **4.55** | **4.53** |
| GLU | 179 | **0.87** | **-4.40** | **0.94** | **-4.36** | **7.98** | **2.54** | **7.59** | **2.53** | **1.69** | **-3.53** |
| GLU | 251 | **22.12** | **21.69** | **22.17** | **21.76** | **22.10** | **21.98** | **22.07** | **21.95** | **22.10** | **21.67** |
| GLU | 262 | **11.56** | **10.72** | **11.37** | **10.61** | **11.56** | **11.19** | **11.51** | **11.14** | **11.54** | **10.68** |
| GLU | 276 | **5.59** | **1.53** | **5.65** | **1.55** | **4.98** | **3.56** | **4.93** | **3.49** | **5.58** | **1.50** |
| GLU | 281 | **4.39** | **4.34** | **4.39** | **4.34** | **4.39** | **4.35** | **4.39** | **4.35** | **4.39** | **4.34** |
| GLU | 286 | **3.96** | **3.94** | **3.96** | **3.94** | **3.96** | **3.95** | **3.96** | **3.95** | **3.96** | **3.94** |
| HIS | 40 | **5.09** | **4.97** | **5.10** | **4.98** | **5.11** | **5.04** | **5.10** | **5.03** | **5.09** | **4.97** |
| HIS | 41 | **5.82** | **5.78** | **5.82** | **5.78** | **5.82** | **5.80** | **5.82** | **5.80** | **5.82** | **5.78** |
| HIS | 63 | **3.21** | **2.58** | **--** | -- | **3.26** | **2.97** | **3.20** | **2.90** | **3.17** | **2.54** |
| HIS | 99 | **4.67** | **4.63** | **4.68** | **4.64** | **4.67** | **4.65** | **4.67** | **4.65** | **4.67** | **4.63** |
| HIS | 145 | **6.18** | **6.17** | **6.18** | **6.17** | **6.18** | **6.18** | **6.18** | **6.18** | **6.18** | **6.17** |
| HIS | 150 | **6.12** | **6.11** | **6.13** | **6.11** | **6.12** | **6.12** | **6.12** | **6.12** | **6.12** | **6.11** |
| HIS | 152 | **5.65** | **5.60** | **5.65** | **5.60** | **5.64** | **5.63** | **5.64** | **5.62** | **5.65** | **5.60** |
| HIS | 154 | **5.88** | **5.83** | **5.88** | **5.83** | **5.87** | **5.86** | **5.87** | **5.85** | **5.88** | **5.83** |
| HIS | 175 | **6.32** | **1.78** | **6.38** | **1.83** | **6.04** | **0.18** | **5.96** | **0.11** | **6.46** | **1.91** |
| HIS | 202 | **2.84** | **-3.25** | **2.91** | **-3.20** | **2.73** | **-3.20** | **2.52** | **-3.53** | **3.35** | **-2.76** |
| HIS | 216 | **5.35** | **5.32** | **5.35** | **5.32** | **5.35** | **5.33** | **5.34** | **5.32** | **5.34** | **5.31** |
| CYS | 11 | **14.53** | **13.17** | **14.47** | **13.14** | **15.34** | **15.16** | **15.37** | **15.14** | **13.96** | **12.60** |
| CYS | 22 | **14.41** | **13.31** | **14.42** | **13.33** | **14.51** | **14.33** | **14.47** | **14.26** | **14.25** | **13.17** |
| CYS | 28 | **12.00** | **11.74** | **12.01** | **11.74** | **12.05** | **11.93** | **12.04** | **11.91** | **11.98** | **11.71** |
| CYS | 50 | **11.76** | **11.21** | **11.78** | **11.22** | **11.77** | **11.35** | **11.76** | **11.34** | **11.77** | **11.22** |
| CYS | 126 | **14.46** | **14.30** | **14.47** | **14.31** | **14.43** | **14.39** | **14.43** | **14.38** | **14.46** | **14.30** |
| CYS | 127 | **12.74** | **12.47** | **12.75** | **12.49** | **12.69** | **12.61** | **12.68** | **12.61** | **12.74** | **12.47** |
| CYS | 196 | **11.47** | **10.81** | **11.47** | **10.81** | **11.83** | **11.57** | **11.81** | **11.52** | **11.36** | **10.69** |
| CYS | 294 | **12.21** | **11.05** | **12.24** | **11.07** | **12.27** | **11.41** | **12.26** | **11.39** | **12.23** | **11.06** |
| TYR | 102 | **10.30** | **10.30** | **10.30** | **10.30** | **10.30** | **10.30** | **10.30** | **10.30** | **10.30** | **10.30** |
| TYR | 104 | **10.27** | **10.25** | **10.27** | **10.26** | **10.27** | **10.26** | **10.27** | **10.26** | **10.27** | **10.25** |
| TYR | 271 | **14.70** | **14.20** | **14.74** | **14.22** | **14.66** | **14.37** | **14.65** | **14.36** | **14.71** | **14.20** |
| LYS | 8 | **-3.30** | **-3.81** | **-3.28** | **-3.79** | **-3.15** | **-3.07** | **-3.28** | **-3.22** | **-3.46** | **-3.97** |
| LYS | 30 | **9.57** | **9.54** | **9.57** | **9.54** | **9.57** | **9.56** | **9.57** | **9.56** | **9.57** | **9.53** |
| LYS | 78 | **11.38** | **11.37** | **11.38** | **11.37** | **11.38** | **11.38** | **11.38** | **11.38** | **11.38** | **11.37** |
| LYS | 160 | **10.30** | **10.29** | **10.30** | **10.29** | **10.30** | **10.29** | **10.30** | **10.29** | **10.30** | **10.29** |
| LYS | 203 | **10.31** | **6.69** | **10.34** | **6.74** | **--** | -- | -- | -- | ***12.43** | ***9.43** |
| LYS | 291 | **9.84** | **9.74** | **9.85** | **9.74** | **9.84** | **9.76** | **9.84** | **9.76** | **9.84** | **9.73** |
| ARG | 38 | **11.12** | **11.04** | **11.12** | **11.04** | **11.13** | **11.10** | **11.12** | **11.10** | **11.11** | **11.03** |
| ARG | 42 | **12.56** | **12.54** | **12.56** | **12.54** | **12.56** | **12.55** | **12.56** | **12.55** | **12.56** | **12.54** |
| ARG | 46 | **12.16** | **12.05** | **12.17** | **12.06** | **12.16** | **12.09** | **12.16** | **12.08** | **12.16** | **12.05** |
| ARG | 84 | **12.46** | **12.46** | **12.46** | **12.46** | **12.46** | **12.46** | **12.46** | **12.46** | **12.46** | **12.46** |
| ARG | 89 | **12.35** | **12.35** | **12.35** | **12.35** | **12.35** | **12.35** | **12.35** | **12.35** | **12.35** | **12.35** |
| ARG | 164 | **11.85** | **11.81** | **11.85** | **11.81** | **11.84** | **11.84** | **11.84** | **11.83** | **11.84** | **11.81** |
| ARG | 213 | **11.00** | **10.86** | **11.00** | **10.86** | **11.00** | **10.92** | **10.99** | **10.90** | **11.00** | **10.84** |
| ARG | 222 | **9.85** | **9.79** | **9.85** | **9.79** | **9.85** | **9.85** | **9.84** | **9.84** | **9.84** | **9.78** |
| ARG | 254 | **12.45** | **12.43** | **12.45** | **12.44** | **12.45** | **12.45** | **12.45** | **12.45** | **12.45** | **12.43** |
| ARG | 280 | **11.82** | **11.74** | **11.83** | **11.74** | **11.82** | **11.76** | **11.82** | **11.76** | **11.83** | **11.74** |
