# Supplementary material for: Unraveling the structural elements of pH sensitivity and substrate binding in the human zinc transporter SLC39A2 (ZIP2)
Source: J Biol Chem. 2020 Aug 26;294(20):8046–63. doi: 10.1074/jbc.RA118.006113 (PMC6527156; doi:10.1074/jbc.RA118.006113)
Supplement: Supporting Information [file supp_294_20_8046__index.html]

Unraveling the structural elements of pH sensitivity and substrate binding in the human zinc transporter SLC39A2 (ZIP2) — Structural basis of transport in SLC39A2 — Unraveling the structural elements of pH sensitivity and substrate binding in the human zinc transporter SLC39A2 (ZIP2) — Structural basis of transport in SLC39A2 — Supporting Information 

# Unraveling the structural elements of pH sensitivity and substrate binding in the human zinc transporter SLC39A2 (ZIP2)

## Supporting Information

- Supporting Information (to be published online) - Alignements and pKa calculations
